# The CAGE Framework (Calcium-Gated Excitability): From Single Neurotransmitters to Calcium States in the Tumor Microenvironment

**DOI:** 10.3390/ijms27177937

**Published:** 2026-09-06

**Authors:** Lena M. Rudy, Michał M. Godlewski

**Affiliations:** 1 Scientific Society of Veterinary Medicine Students, Warsaw University of Life Sciences—SGGW, Nowoursynowska 159, 02-776 Warsaw, Poland; 2 Department of Physiological Sciences, Warsaw University of Life Sciences—SGGW, Nowoursynowska 159, 02-776 Warsaw, Poland

**Keywords:** cancer neuroscience, tumor microenvironment, calcium signaling, nicotinic acetylcholine receptors, GABAergic signaling, α2δ-1, *CACNA2D1*, Calcium-Gated Excitability

## Abstract

The tumor microenvironment (TME) is an innervated and neurochemically active setting. To date, its cholinergic and GABAergic signaling have largely been analyzed as separate systems, contributing to inconsistent findings across the literature. We propose a Calcium-Gated Excitability (CAGE) model, in which α7 nicotinic acetylcholine receptor (nAChR), α4β2 nAChR, GABA type A (GABA_A_) and type B (GABA_B_) receptors, and α2δ-1 converge on a single downstream variable: Ca^2+^ flux. α7 conducts Ca^2+^ directly; α4β2 may redirect cholinergic input toward GABA release; GABA_A_ and GABA_B_ set the direction of the calcium response through intracellular chloride-gradient state and opposing intracellular signaling branches; α2δ-1 sets the gain that determines whether depolarization reaches a functional threshold. The resulting calcium-axis configuration, defined by receptor functional state, chloride-gradient status, α2δ-1 gain, and ligand exposure, shapes Ca^2+^ output and constrains downstream biological responses. Under this framework, some divergent findings on acetylcholine, nicotine, and GABA become testable consequences of distinct calcium-regulatory configurations. CAGE yields testable predictions, including nonlinear nAChR dose–response curves, reversal of GABA_A_ polarity, α2δ-1-dependent signal scaling, and spatially heterogeneous Ca^2+^ states. Studies evaluating nAChR- and GABA-receptor-targeted therapies without prior calcium-axis stratification may not be underpowered; they may be asking the wrong question.

## 1. Introduction

Tumors develop within a dynamically evolving tumor microenvironment (TME), the complexity of which extends far beyond the autonomous properties of cancer cells themselves [1,2]. The TME encompasses immune and stromal components, including T lymphocytes, tumor-associated macrophages, cancer-associated fibroblasts (CAFs), endothelial cells, and pericytes, which through the secretion of cytokines, growth factors, and extracellular matrix-remodeling enzymes co-regulate proliferation, angiogenesis, and treatment response [1,2,3,4]. Accumulating evidence indicates that the TME simultaneously functions as an active site of neurochemical regulation: both tumor innervation and tumor and stromal cells themselves provide sources of neurotransmitters that act in an autocrine and paracrine manner on receptors aberrantly expressed in tumors [5,6,7]. Despite this, the dominant research approach is to analyze each individual neurotransmitter system in isolation, without embedding it within a common effector framework [7,8,9,10]. A systemic account of how neurochemical cues converge on a common central biological integrator, namely calcium ions (Ca^2+^), is still lacking. Calcium-Gated Excitability (CAGE) posits that cellular excitability within the TME is shaped through neurochemical gating of Ca^2+^ regulation, rather than generated by calcium per se.

At the intracellular level, Ca^2+^ ions function as a central second messenger integrating diverse surface inputs into specific biological decisions: activation of the cell cycle through cyclins and Ca^2+^/calmodulin-dependent kinases, cytoskeletal reorganization and migration dependent on calpains and Rho GTPases, as well as expression of proangiogenic genes through the hypoxia-inducible factor 1 alpha (HIF-1α) and nuclear factor kappa B (NF-κB) pathways [11,12,13,14]. Disturbances in the amplitude and dynamics of Ca^2+^ transduction lead to the stabilization of a proliferative phenotype, increased invasiveness, and resistance to apoptosis, thereby reinforcing core malignant phenotypes [11,12,15]. Ca^2+^ influx and intracellular Ca^2+^ dynamics in TME cells are regulated by multiple systems, including voltage-gated calcium channels (VGCCs), transient receptor potential (TRP) family channels, store-operated Ca^2+^ entry mediated by stromal interaction molecule (STIM) and Orai proteins, and Ca^2+^ release from intracellular stores through inositol 1,4,5-trisphosphate (IP_3_) and ryanodine receptors [11,16,17,18]. Under TME conditions, channel activity is shaped by neurochemical, ionic, and spatial constraints, allowing the same Ca^2+^ machinery to support divergent biological outputs [11,15,17].

Among the neurotransmitter systems present within the TME, the cholinergic and GABAergic systems are particularly well positioned to regulate calcium homeostasis through direct and coupled mechanisms [8,9]. The α7 nAChR, characterized by high Ca^2+^ permeability, constitutes a potentially efficient pro-calcium entry pathway that activates the phosphoinositide 3-kinase/protein kinase B (PI3K/AKT), mitogen-activated protein kinase/extracellular signal-regulated kinase (MAPK/ERK), and Janus kinase/signal transducer and activator of transcription (JAK/STAT) pathways [9,19,20,21]; simultaneously, heteromeric α4β2 nAChRs, best characterized as presynaptic regulators of neurotransmitter release in the nervous system, may constitute a coupling point between cholinergic and GABAergic communication under TME conditions [9,22]. GABAergic transmission is not a simple “inhibitory system”: GABA_A_ receptors modulate membrane potential and indirectly voltage-gated Ca^2+^ channel activity, whereas GABA_B_ receptors, through inhibitory G proteins (G_i/o_), directly restrict Ca^2+^ influx through voltage-gated calcium (Cav) 2.x channels [8,23,24]. As a result, the direction of the GABA_A_ effect depends critically on the architecture of the chloride gradient in any given cell [25,26]. The third component of the system, the α2δ-1 subunit of voltage-gated calcium channels (*CACNA2D1*), acts not merely as an auxiliary protein, but as a regulator of Ca^2+^ response gain, thereby determining cellular calcium competence, namely the ability to translate a neurochemical stimulus into a biologically effective response, while also serving as a marker of cancer stem cells (CSC) in multiple solid tumors [27,28,29,30].

Together, these components position excitation-inhibition balance as a variable that links receptor activation, channel availability, and local ionic conditions to the amplitude and direction of the Ca^2+^ response [8,9,30]. Functionally, the α7 nAChR serves as a generator of a pro-calcium drive, α4β2 nAChR as a potential node coupling cholinergic signaling with GABA, GABA_A_ and GABA_B_ receptors as contextual modulators of the direction of the calcium response, and the α2δ-1 subunit as a regulator of response amplitude and cellular transduction competence [10,22,30,31]. A pro-excitatory configuration, characterized by α7-associated pro-calcium signaling and high α2δ-1 gain, is expected to favor amplified Ca^2+^ responses, whereas a buffered configuration, associated with preserved GABAergic restraint and hyperpolarizing chloride handling, is expected to limit them [23,25,30,32,33]. A separate rewired configuration is considered when downstream oncogenic or immune signaling modifies the biological consequence of proximal neurochemical Ca^2+^ regulation, as described below for GABA_B_-dependent signaling [34,35]. This distinction separates calcium-regulatory state from downstream phenotypic interpretation and provides the rationale for the analysis that follows [8,9,31,34].

The physical interstitial environment adds a spatial layer to this regulation [36,37]. Abnormal tumor angiogenesis leads to elevated interstitial fluid pressure (IFP), impaired lymphatic drainage, and the dominance of diffusion over convection as the mechanism of molecular transport [37,38,39,40]. Consequently, given the diffusion-limited transport characteristic of solid tumors, neurotransmitters locally secreted by TME cells, including acetylcholine (ACh) from cholinergic T lymphocytes and other non-neuronal TME components, potentially including stromal cells and CAFs, and GABA from B cells and macrophages, are expected to generate spatially heterogeneous exposure fields rather than a homogeneous ligand distribution, potentially differentiating calcium responses among cell subpopulations within a single tumor [35,36,41,42,43,44,45]. The Ca^2+^ output in the TME is therefore a function not only of receptor expression and ligand availability, but also of their spatial organization and exposure time, making the interstitial environment an active spatial regulator that can differentiate calcium responses across tumor regions in addition to differences arising from the molecular profile of individual cells [36,38,46,47].

CAGE is used here to integrate cholinergic transmission, GABAergic signaling, and α2δ-1-dependent channel regulation into a single mechanistic sequence. The review therefore first evaluates the experimental evidence for each component separately before considering whether their combined behavior can be organized into a common calcium-centered framework. Each component is considered according to the specific step it controls: activation onset, directional modulation, response gain, or spatial exposure. This organization provides a basis for the mechanistic sections that follow and for experimental stratification of neurochemical Ca^2+^ responses across distinct TME contexts [7,8,30,34].

## 2. Neurochemical Axis of Ca^2+^ Regulation in the Tumor Microenvironment

**Figure 1:**
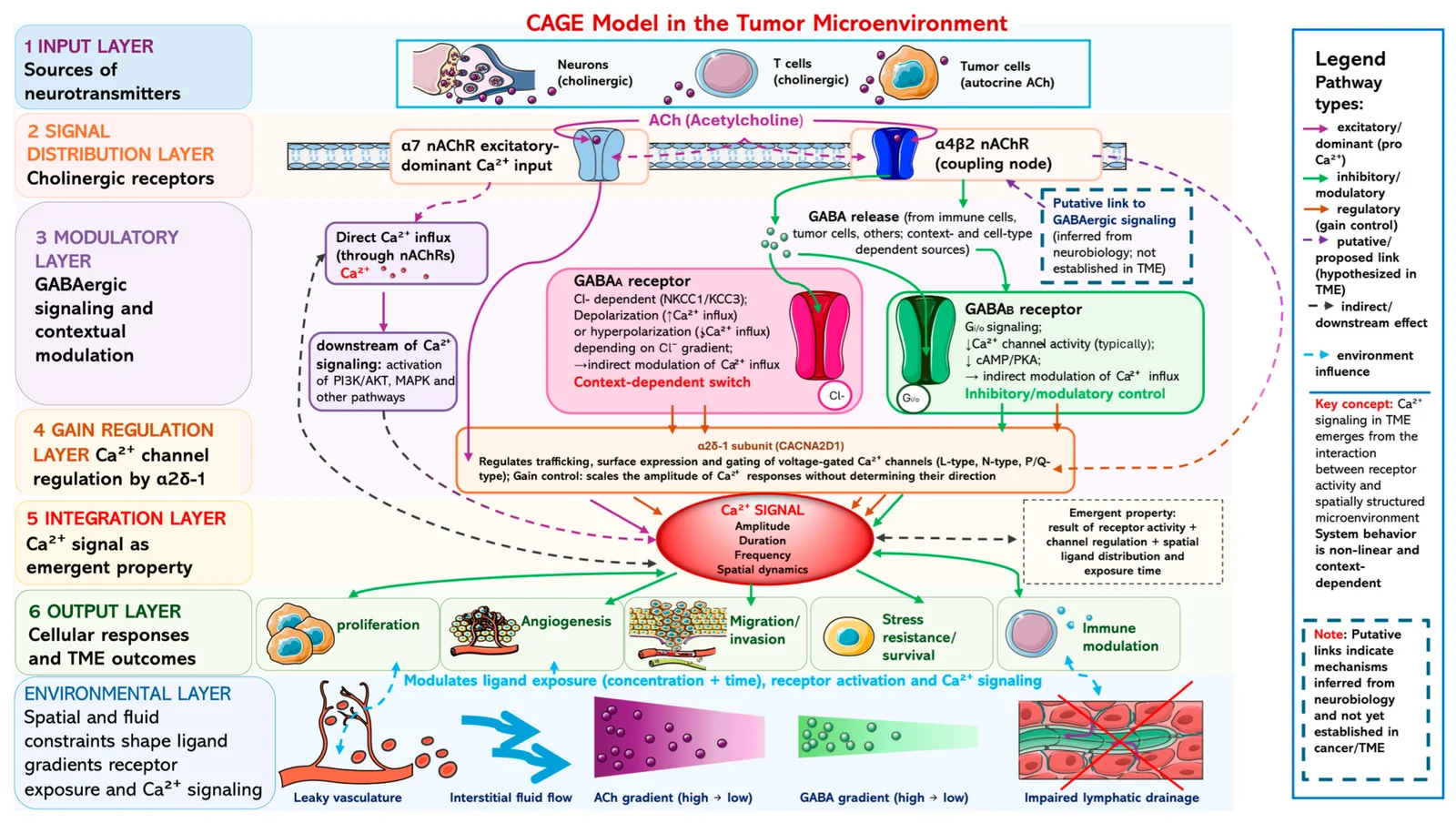
**The CAGE Model: Neurochemical Control of Ca^2+^ Dynamics in the Tumor Microenvironment.** The CAGE model conceptualizes Ca^2+^ regulation in the tumor microenvironment (TME) as an emergent property of an interconnected neurochemical system. Within this architecture, α7 nAChR provides a multimodal pro-calcium drive through ionotropic and intracellular signaling mechanisms, whereas α4β2 nAChR may couple cholinergic activity to the GABAergic axis. GABA_A_ and GABA_B_ receptors determine the direction and magnitude of the calcium response through effects on membrane potential, chloride homeostasis, and G_i/o_-dependent channel inhibition. The α2δ-1 subunit further defines cellular calcium competence by controlling channel availability and response gain. These receptor- and channel-level mechanisms are superimposed on spatial constraints imposed by TME interstitial fluid transport, vascular permeability, and defective lymphatic drainage, which shape local neurotransmitter gradients and exposure duration. The integrated configuration primarily shapes the calcium-regulatory output of a given TME niche, while downstream oncogenic and immune signaling contributes to its biological consequence. Abbreviations: CAGE, Calcium-Gated Excitability; TME, tumor microenvironment; nAChR, nicotinic acetylcholine receptor; GABA_A_/GABA_B_, γ-aminobutyric acid type A/type B receptor; G_i/o_, inhibitory G-protein family. Graphical elements from Servier Medical Art.

The classical mechanisms regulating Ca^2+^ influx in cancer cells are VGCCs, channels of the TRP family, the store-operated calcium entry (SOCE) mechanism mediated by STIM and Orai proteins, as well as the release of Ca^2+^ from intracellular stores through IP_3_ and ryanodine receptors. Together, these pathways constitute the canonical calcium architecture of the TME [11,12,15,17,48]. However, increasing evidence indicates that an additional, partially autonomous subsystem is involved in the control of this architecture: a neurochemical layer in which locally acting neurotransmitters modulate calcium-channel activity through membrane potential, ionotropic receptor activation, and regulation of Ca^2+^ channel availability at the plasma membrane [7,8,27,30]. This system does not replace classical channels but rather introduces an additional level of control dependent on the local neurochemical composition of the microenvironment. This layer influences how the canonical calcium machinery is recruited under local TME conditions. The integrated architecture of the proposed system is summarized in Figure 1.

A key distinction from classical receptor signaling is that neurotransmitters within the TME act in an environment that also produces them [5,7,43]. Sources of acetylcholine are not limited to tumor-infiltrating nerve fibers; numerous TME cells exhibit expression of elements of the non-neuronal cholinergic system, including choline acetyltransferase (ChAT), the vesicular acetylcholine transporter (VAChT), and the choline transporter, particularly in endothelial cells and immune cells, such as T lymphocytes and tumor-associated macrophages (TAM) [42,43,44,49]. Of particular significance are cholinergic T lymphocytes, a newly described population mediating communication between the nervous system and the immune response within the TME, whose dysfunction may affect the efficacy of immunotherapy [44,50]. The GABAergic component has similarly diverse cellular sources: B lymphocytes and macrophages can synthesize and secrete GABA, indicating that neurochemical calcium regulation is not restricted to neurons but emerges from a complex, immunologically active network of cells within the tumor niche [35,45]. Consequently, both acetylcholine and GABA may act in an autocrine and paracrine manner, activating aberrantly expressed receptors in cancer and TME cells and influencing proliferation, migration, invasion, angiogenesis, and the immune response [7,8,21,35].

Mechanistically, cholinergic and GABAergic signaling regulate Ca^2+^ influx through complementary routes. The first is direct: nAChRs, particularly α7, conduct Ca^2+^ as ion channels. The second is indirect: cholinergic and GABAergic signaling modulate membrane potential and thereby VGCC activity, altering intracellular Ca^2+^ concentration [8,19,23,51]. The third component concerns the availability of Ca^2+^ channels themselves: the α2δ-1 subunit of voltage-gated calcium channels, which is the pharmacological target of pregabalin and gabapentin. It controls the trafficking of channels to the cell membrane and their functional efficiency, thereby influencing the efficiency with which receptor activation is translated into a calcium response [27,52,53]. Consequently, regulation occurs at three linked levels: α7 nAChR-mediated activation, directional modulation by GABAergic transmission, and response gain through α2δ-1. Because these levels can operate within the same TME compartment, the following sections analyze them as linked steps: cholinergic input generation, GABAergic directional control, and α2δ-1-dependent response gain [7,8,19,27,30].

### 2.1. Calcium Architecture and Transduction Competence

Remodeling of Ca^2+^ dynamics is among the best-documented mechanisms driving tumor progression [11,12]. What carries biological information is not the absolute ion concentration but the spatiotemporal pattern of the response, its amplitude, duration, and frequency, which specifies distinct cellular decisions: cell-cycle entry, migration, angiogenesis, or resistance to stress and treatment [11,12,15]. This spatiotemporal code is the level at which neurochemical inputs can reconfigure calcium-dependent behavior.

At the molecular level, Ca^2+^ influx is regulated by four principal systems [11,17]. VGCCs (mainly L- and N-type) undergo aberrant expression in many solid tumors and participate in mechanistic target of rapamycin complex 2 (mTORC2)/AKT-dependent proliferation; however, their effects are context-dependent, as overexpression of L-type VGCCs in breast and prostate cancer can be oncogenic, whereas in other tissues these channels may serve suppressive functions [18,54,55]. TRP channels (TRPV1, TRPV4, TRPC1, TRPC5, TRPM2, TRPV6) exhibit differential overexpression across various cancer types and modulate proliferation, migration, drug resistance, and inflammatory response through ERK1/2, PI3K/AKT, and NF-κB cascades [56,57]. The SOCE mechanism, mediated by the STIM1-Orai1 complex, constitutes one of the principal pathways of Ca^2+^ influx in non-excitable cells, including cancer cells, and participates in the regulation of proliferation, invasion, angiogenesis, and resistance to apoptosis [16,48,58]. The Orai3 isoform, frequently described as overexpressed in prostate cancer and non-small cell lung cancer (NSCLC), forms heteromers with Orai1, reducing the classical calcium release-activated calcium (CRAC) current and increasing resistance to apoptosis [59,60,61,62]. It should, however, be noted that the role of Orai3 remains context-dependent across cancer types and experimental systems. In NSCLC, available cell-line data indicate that Orai3 supports SOCE and proliferation, whereas the relative contribution of Orai3 to apoptosis resistance and other cancer phenotypes varies among tumor contexts [59,60]. For prostate cancer the consensus is stronger (data from LNCaP lines and in vivo models) than for NSCLC, where the literature remains fragmentary [59,61,62]. The fourth system, the release of Ca^2+^ from the endoplasmic reticulum (ER) through IP_3_ and ryanodine receptors, couples surface signaling with intracellular ion stores and participates in calcium-signal amplification [11,12,63]. CAFs exhibit their own reprogramming of this architecture through L-VGCC, TRPC3, and SOCE, thereby regulating their differentiation, migration, and genetic instability after irradiation, indicating that remodeling of the Ca^2+^ axis is not limited to cancer cells but encompasses the entire cellular ecosystem of the TME [64]. Together, these four systems define the channel-level substrate on which neurochemical regulation operates.

The key conclusion is that calcium-channel output is not determined by channel identity alone, but by the environmental conditions in which channels are activated [16,59]. In lung cancer cells, activation of SOCE leads to sustained Ca^2+^ influx directly driving proliferation, whereas silencing of Orai3 lowers basal Ca^2+^ concentration and inhibits cell growth [60,61]. These effects are strongly modulated by the ionic and metabolic environment of the TME [16,59]. This observation supports a systems-based interpretation: the same channel can generate divergent outcomes when engaged under different environmental conditions. Such heterogeneity underscores the context-dependent relationship between aberrant Ca^2+^ signaling and cancer progression.

The TME is physiologically distinct from normal tissues in ways that directly affect calcium-channel activity [65]. The acidic tumor microenvironment, driven in part by aerobic glycolysis and lactate production, modulates Ca^2+^-permeable channels and contributes to the selection of aggressive tumor phenotypes [65,66,67,68]. Tumor extracellular pH is spatially heterogeneous and is commonly reported in an acidic range of approximately 6.5–7.0, with lower values documented in selected tumor regions [65,69]. Importantly, aerobic glycolysis can occur despite adequate oxygen availability, whereas hypoxia provides an additional source of metabolic and physicochemical stress [65,70]. Consequently, pH-dependent modulation of calcium signaling need not be confined to severely hypoxic tumor regions [65,66,67]. Thus, TME physicochemistry constitutively modifies Ca^2+^-channel responsiveness and must be treated as a baseline condition for neurochemical regulation [65,71].

Neurochemical modulation of Ca^2+^ influx requires three conditions: ligand availability, receptor expression, and cellular capacity to translate receptor activation into altered calcium entry [71]. These conditions are fulfilled within the TME both neuronally and non-neuronally, as described in detail in the following sections, but their realization depends on the current calcium architecture of a given cell: which channels are expressed, in what state of activation they exist, and how the ionic environment and pH modify their responsiveness [17,65,71].

### 2.2. Cholinergic Regulation of Ca^2+^ Dynamics in the Tumor Microenvironment

Cholinergic activity in the TME has two separable calcium-relevant functions: direct Ca^2+^ entry through α7 nAChR and potential redirection of the signal through α4β2-linked GABAergic modulation [10,72,73,74].

Acetylcholine in the TME originates from multiple sources. In non-small cell lung cancer (NSCLC), A549, H1299, and H1975 cells express choline acetyltransferase (ChAT) as part of a broader cholinergic machinery that enables lung cancer cells to synthesize and secrete ACh. This locally produced ACh can act as an autocrine growth signal through nicotinic and muscarinic acetylcholine receptors [75,76,77,78,79]. An analogous mechanism has been described in pancreatic cancer (Panc-1) and colorectal cancer (HT29), where nAChR blockade reduces the rate of cell growth [21,80,81]. In parallel, acetylcholine is supplied by immune cells—ChAT-expressing T lymphocytes, indicating that cholinergic input may be relatively continuous and locally amplified, independently of tumor innervation [44,50]. Thus, the magnitude of the cholinergic drive reflects the activity of a neurotransmitter-producing cellular network, not innervation density alone, limiting the interpretability of interventions directed exclusively at nerve fibers [44,75,82].

α7 nAChR functions as a homopentameric cation channel with high permeability to Ca^2+^. Importantly, the high Ca^2+^ permeability of α7, described as a P_Ca_/P_Na_ ratio of approximately 10–20, is a property of the physiological receptor variant and may undergo substantial modification in cancer cells [19,51]. In cancers, alternative variants of *CHRNA7* transcripts have been described, including the human-specific *CHRFAM7A* duplication [83,84,85]. The truncated dupα7 subunit encoded by *CHRFAM7A* does not form functional homopentameric channels on its own, and previous characterizations across neuronal and inflammatory contexts have described it as a dominant-negative modulator that can reduce functional α7 surface expression and signaling [83,84]. The ligand-specific consequences of dupα7 are particularly relevant to α7 modulation by secreted Ly6/uPAR-related protein 1 (SLURP-1), an endogenous auto/paracrine epithelial regulator that acts as a negative allosteric modulator of α7 nAChR [86]. Recent electrophysiological data from metastatic melanoma indicate that the effect of dupα7 is not uniformly dominant-negative: α7 and dupα7 subunits can co-assemble into functional hybrid α7/dupα7 channels whose nicotine potency is not significantly different from that of homomeric α7 nAChRs, while their sensitivity to recombinant SLURP-1 is reduced; notably, the synthetic SLURP-1-derived peptide Oncotag retains similar inhibitory efficacy on both receptor forms [87]. High *CHRFAM7A*/dupα7 expression in this context was associated with resistance of metastatic melanoma cells to recombinant SLURP-1-mediated inhibition, indicating that receptor subunit composition can determine ligand-specific responses to α7-targeted modulation [87]. These findings indicate that the functional consequence of α7 expression in the TME depends not only on transcript abundance but also on receptor subunit composition and on the specific ligand acting on the receptor—agonist or modulator—rather than on a uniform gain or loss of α7 function [83,85,87]. Accordingly, *CHRNA7* expression alone is not a sufficient stratification parameter, and characterization of *CHRFAM7A*/dupα7 should be incorporated when comparing α7-dependent signaling or responses to α7-targeted interventions across cancer models [83,84,85,87].

The oncological relevance of this extracellular regulatory layer is supported by experimental studies of SLURP-1 and its synthetic derivatives. In A431 epidermoid carcinoma cells, recombinant SLURP-1 suppresses proliferation through an α7-dependent mechanism and alters multiple mitogenic signaling pathways, while in A431 xenografts systemic administration of SLURP-1 suppresses primary tumor growth and metastatic progression [86]. More recent in vivo work further showed that SLURP-1 can enhance the antitumor and antimetastatic effects of low-dose doxorubicin in the same xenograft system, supporting the preclinical therapeutic relevance of α7-directed endogenous modulation [88]. A synthetic peptide derived from the active loop of SLURP-1, Oncotag, likewise suppresses tumor growth in vivo and produces more sustained inhibition of several pro-oncogenic signaling pathways [86]. These findings demonstrate that endogenous-like allosteric regulation of α7 can modify oncological output independently of changes in receptor abundance and establish α7 regulatory ligands as an additional determinant of the functional cholinergic state. Importantly, full-length SLURP-1 can also interact with EGFR, whereas Oncotag displays greater α7 selectivity, indicating that the biological effect of an endogenous α7 regulator may additionally depend on receptor-complex context rather than α7 occupancy alone [86].

Beyond its ionotropic function as a Ca^2+^-permeable channel, α7 nAChR can also engage metabotropic intracellular signaling. Metabotropic α7 signaling can extend beyond the brief channel-open state and persist in receptor conformations that are ionotropically desensitized, indicating that loss of sustained ionic conductance does not necessarily terminate α7-dependent intracellular signaling [89,90]. Activation of α7 initiates rapid Ca^2+^ influx, generating local calcium microdomains that may subsequently be amplified by secondary Ca^2+^ release from the ER [19,72,91]. In neuronal models, α7 activation has been shown to trigger both calcium-induced calcium release (CICR) and G-protein-dependent Gα_q_/PLC/IP_3_R signaling, providing ionotropic and metabotropic routes, respectively, for amplification of the calcium signal beyond the receptor channel itself [72,89,90,92,93,94]. These mechanisms have been documented primarily in neuronal preparations, including hippocampal and dorsal root ganglion systems [92,95,96]. In dorsal root ganglion neurons, intracellular Ca^2+^-release machinery involving RyR and IP_3_R is concentrated within subplasmalemmal ER domains, providing an anatomical basis for coupling membrane signaling to intracellular Ca^2+^ stores. Extrapolation to cancer cells is biologically plausible, given their extensive ER and functional IP_3_ receptors, but should be treated as a working hypothesis rather than an established mechanistic link. Furthermore, α7 activation causes membrane depolarization, which may secondarily open L-type voltage-gated calcium channels, increasing total Ca^2+^ influx beyond the component conducted directly through the receptor itself [18,72]. Ca^2+^ influx activates calmodulin, Ca^2+^/calmodulin-dependent kinases, and calpain, which regulate cytoskeletal remodeling, adhesion, and cell migration [13,97,98]. Potential amplification through CICR and L-VGCCs means that α7 may function as a trigger of a nonlinear calcium response, particularly in cells with high α2δ-1 expression, where the availability of L-type channels is increased; this remains to be verified in oncological models.

α7-dependent calcium transduction has been documented in multiple tumor types [9,10,21]. In NSCLC cells (A549, H1299), exposure to nicotine or the tobacco-specific carcinogen 4-(methylnitrosamino)-1-(3-pyridyl)-1-butanone (NNK) leads to an α7-dependent increase in Ca^2+^ concentration, abolished by α-bungarotoxin or *CHRNA7* knockdown, with activation of the PI3K/AKT and MAPK/ERK pathways and increased cyclin D1 expression [20,33,99]. In colorectal cancer (HT29, DLD-1), α7 activation by NNK induces epithelial–mesenchymal transition (EMT) with decreased E-cadherin and increased expression of Snail and ZEB1 [80,100,101]. In breast cancer (MCF-7), direct coupling of α7 nAChR to heterotrimeric G proteins provides an oncological example of this metabotropic signaling mode: interactions with Gα_i/o_ and Gα_q_ enhance proliferation, motility, and calcium signaling, while nicotine-associated α7 signaling has also been linked to changes in the balance between Bax and Bcl-2 and to chemoresistance, whereas α7-dependent STAT3/NF-κB signaling has so far been documented mainly in colorectal and lung cancer models [20,102,103,104]. The α7 nAChR is also expressed in endothelial cells of the TME: in human umbilical vein endothelial cells (HUVECs), α7 activation increases Ca^2+^ influx, proliferation, and the formation of capillary-like structures, particularly under hypoxia, where α7 expression is elevated [105,106]. Conversely, α7 blockade in vivo reduces vessel density within the tumor [105,106]. α7 expression has also been identified in T lymphocytes and tumor-associated macrophages (TAMs), where receptor activity has been linked to modulation of the JAK2/STAT3 pathway [104,107]. Collectively, these data position α7 nAChR as a multimodal signaling node whose output can involve ionotropic Ca^2+^ conductance or metabotropic intracellular signaling, with the relative contribution and biological consequence of these modes varying across tumor, vascular, and immune compartments of the TME.

α4β2 nAChR serves a different function: it is not primarily a Ca^2+^-conducting channel, but rather a presynaptic regulator of neurotransmitter release [19,74]. In the nervous system, activation of α4β2 on GABAergic terminals increases GABA release through local depolarization of the terminal, secondary opening of Cav2.2 and Cav2.1 channels, elevation of Ca^2+^ concentration in the terminal and exocytosis of GABA-containing vesicles [73,108,109]. This effect is abolished by the antagonist dihydro-β-erythroidine [108]. For α4β2, the strongest functional data concern its presynaptic localization on neuronal terminals and GABAergic interneurons. The role of this receptor in cancer cells themselves remains far less documented than for α7 [9,73]. A key methodological caveat is that all mechanistic data on α4β2-mediated modulation of GABA derive from neuronal preparations, including hippocampal and cortical interneuron models, and have not yet been directly replicated in cancer cells or in vivo TME models [74,109,110]. Cancer cells express α4β2 nAChR, but their subcellular architecture, absence of classical presynaptic terminals, and distinct repertoire of Cav2.x channels may substantially modify or abolish this mechanism relative to its neuronal form [9,10]. The α4β2-GABA link in the TME should therefore be treated as a biologically justified, testable hypothesis rather than an established oncological mechanism. Its importance lies in the possibility that α4β2 redirects cholinergic input toward GABA-dependent modulation rather than direct α7-dominated Ca^2+^ entry.

Nevertheless, oncological data support a functional interaction between nicotinic and GABAergic transmission. In NNK-induced lung adenocarcinoma models and in NSCLC cells (NCI-H322), nicotinic receptor activation simultaneously regulates two opposing axes: α7-dependent enhancement of excitatory drive (noradrenaline, cyclic adenosine monophosphate [cAMP]) and α4β2-dependent modulation of GABA [111,112]. Acute nicotinic stimulation can enhance GABAergic output through α4β2-containing nAChRs [109], whereas prolonged exposure introduces a substantially more complex receptor-state dependence. At the level of intrinsic receptor kinetics, homomeric α7 nAChRs undergo exceptionally rapid desensitization following agonist activation, while α4β2 nAChRs desensitize more slowly but display substantially greater sensitivity to low nicotine concentrations and may enter long-lived desensitized states during sustained exposure [113]. Chronic nicotine exposure can additionally increase α4β2 surface expression, demonstrating that receptor abundance and functional availability are not equivalent variables [114]. This distinction is particularly important when interpreting oncological studies. In NNK-induced pulmonary and pancreatic adenocarcinoma models, prolonged exposure was associated with suppression of the GABA-synthesizing enzyme GAD65 and reduced GABA levels, together with increased protein expression of both α7 and α4 nAChR subunits and enhanced cAMP-associated signaling [111]. Thus, the experimentally observed shift towards stimulatory signaling cannot be reduced to a simple sequence in which α4β2 becomes desensitized while α7 remains continuously active. Increased receptor abundance may coexist with functional desensitization, and the net output reflects receptor expression, agonist concentration and exposure history, recovery kinetics, and downstream transcriptional and enzymatic regulation [113,114]. Accordingly, chronic nicotinic or NNK exposure is better viewed as remodeling the functional balance between α7-associated pro-calcium signaling and α4β2-linked GABAergic modulation rather than producing an obligatory α7-on/α4β2-off switch. In lung and pancreatic cancer models, the convergent phenotype is reduced inhibitory GABAergic tone together with enhanced stimulatory signaling, but the receptor-level mechanisms generating this state remain exposure- and context-dependent [111,112,115]. Within CAGE, exposure history should therefore be treated as a determinant of receptor functional state rather than as a surrogate for receptor subtype dominance.

Together, these data indicate that exposure history dynamically modifies the functional α7/α4β2 balance and thereby the coupling of cholinergic input to Ca^2+^ and GABAergic output [113,114]. The α4β2 component thus provides the mechanistic transition to GABAergic regulation, where Ca^2+^ output is further shaped by chloride handling, GABA_A_/GABA_B_ receptor subtype, and downstream effector coupling [25,116,117].

### 2.3. Contextual Regulation of Ca^2+^ by GABAergic Modulation in the Tumor Microenvironment

GABAergic signaling in cancers is not a simple “inhibitory counterpart” of excitatory drive. Its calcium-regulatory effect depends on receptor type, chloride-gradient architecture, Ca^2+^-channel repertoire, and the cellular composition of the TME [118,119,120]. This requires distinguishing two levels that directly determine Ca^2+^ regulation: (1) the receptor mechanism, GABA_A_ versus GABA_B_; and (2) the ionic context of the target cell, which determines the direction of the GABA_A_ effect. A separate downstream layer must also be considered, as GABA_B_ signaling can engage autonomous oncogenic or immune pathways whose phenotypic consequences are not reducible to its classical Ca^2+^-inhibitory action.

Activation of the GABA_A_ receptor increases Cl^−^ conductance according to the electrochemical gradient. Under classical conditions this hyperpolarizes the membrane, secondarily reducing the probability of opening of voltage-gated calcium channels and limiting Ca^2+^-dependent activation of proliferative and migratory programs [119,120]. However, this requires low intracellular Cl^−^ concentration, a “mature” chloride phenotype in which Cl^−^ extrusion predominates over accumulation [119,120]. In cancer cells, this condition is often not met. Increased activity of the Na^+^-K^+^-2Cl^−^ cotransporter 1 (NKCC1, encoded by *SLC12A2*) promotes Cl^−^ accumulation in the cytoplasm, shifting the chloride equilibrium potential (E_Cl_) toward more positive values. Consequently, GABA_A_ receptor activation may cease to be hyperpolarizing and instead become depolarizing, secondarily activating voltage-gated Ca^2+^ channels and switching the signal from antiproliferative to pro-growth or pro-invasive [25,121]. A terminological caveat is important here: the direction of GABA_A_ signaling should be inferred from the net chloride gradient rather than from the status of any single transporter. KCC2 (SLC12A5) is the canonical neuronal K^+^/Cl^−^ cotransporter underlying the mature hyperpolarizing GABA phenotype, whereas non-neuronal cancer cells can employ a broader repertoire of Cl^−^-extrusion mechanisms, including KCC1 and KCC3 as well as anion exchangers (AE) and sodium-bicarbonate cotransporters (NBC), whose expression and activity depend on tissue type, proliferative state, and ionic environment [25,26,122,123,124]. The relevant variable is therefore the relative predominance of Cl^−^ accumulation mechanisms, particularly NKCC1, over the available efflux mechanisms, which determines E_Cl_ and thereby the direction of the GABA_A_ response [25]. Thus, chloride-gradient architecture determines the sign of the GABA_A_ effect and should be treated as a model-specific variable.

Consequences of this switch are documented by data from two models. In triple-negative breast cancer (TNBC), HCC1806 and BT-549 cells exhibit increased expression of surface GABA_A_ receptors containing the β3 subunit, functional Cl^−^ flow through these receptors, and direct dependence of proliferation and migration on this axis: pharmacological blockade or β3 knockdown reduced cyclin D1 expression, increased p21, and arrested the cell cycle in G_0_/G_1_, indicating that the chloride receptor acts here as an active regulator of the growth program, not a passive marker [117]. In a model of *BRAF*^600E^-mutant melanoma initiation, GABA synthesized by melanocytic cells mediates functional communication with keratinocytes through a bioelectrical component dependent on chloride conductance. Disruption of this circuit weakened tumor initiation [125,126]. Both models indicate that chloride-dependent GABAergic signaling can be functionally integrated into oncogenic programs; however, the extent to which depolarization-driven Ca^2+^ influx constitutes the causal intermediate remains to be established directly [117,125,126].

The GABA_B_ receptor acts through two effector limbs that must be considered jointly, because they can drive the growth program in opposite directions [116,118]. The G-protein beta-gamma subunit complex (Gβγ) branch directly inhibits Cav2.1 and Cav2.2 channels, reducing Ca^2+^ influx and neurotransmitter release, and could provide a mechanistic basis for an antiproliferative GABA_B_ effect in cancer cells that retain these channels [127,128]. The Gα_i/o_ route inhibits adenylyl cyclase, lowers cAMP, and decreases protein kinase A (PKA) activity, thereby reducing PKA-dependent phosphorylation of Ca^2+^ channels and regulatory proteins [116,118]. The direction of this second route is mechanistically important and is frequently misread: because PKA inhibits glycogen synthase kinase-3β (GSK-3β) and can additionally stabilize β-catenin directly, a GABA_B_-driven fall in cAMP/PKA, taken in isolation, relieves this inhibition, favoring active GSK-3β and accelerated β-catenin turnover, i.e., a growth-restraining outcome [129]. The β-catenin-stabilizing effect attributed to GABA_B_ in some tumors therefore cannot be inferred from the cAMP/PKA route; as developed below, it is produced by a distinct AKT-dependent mechanism [130]. Treating GABA_B_ as a single “presynaptic Ca^2+^ brake,” or inferring its action from Gβγ blockade alone, conflates signaling branches that need not co-vary and may oppose each other in their net effect on proliferation [116,118].

In a range of solid tumors, GABA_B_ is not limited to its classical role as a presynaptic Ca^2+^ brake but can be co-opted by cell-autonomous survival and immune-evasion pathways [34]. This has been best documented in lung cancer and colorectal cancer, where cancer cells with aberrant glutamate decarboxylase 1 (*GAD1*) expression redirect glutamine toward GABA synthesis, secrete GABA in an autocrine manner, and then use signaling through the GABA_B_ receptor to inactivate GSK-3β through inhibitory Ser9 phosphorylation, stabilize β-catenin, raise cyclin D1, and accelerate proliferation [34]. Mechanistically, this Ser9 event reflects PI3K/AKT activity rather than the cAMP/PKA limb: in oncogenically reprogrammed cells, GABA_B_ receptor signaling can engage PI3K/AKT, resulting in AKT-dependent GSK-3β inhibition and β-catenin stabilization [130]. This provides one route by which GABA_B_ signaling may become associated with a pro-proliferative phenotype despite its classical Ca^2+^-inhibitory actions. The available studies therefore indicate that downstream pathway engagement can modify the phenotypic consequence of GABA_B_ activation; however, the variables determining when such rewiring occurs across tumor models remain unresolved [34,130].

GABAergic regulation also operates at the immunological level, shaping tumor survival even when its direct influence on cancer-cell Ca^2+^ dynamics is moderate [35,131]. Activated B cells and plasma cells synthesize and secrete GABA, which promotes differentiation of monocytes and macrophages towards an interleukin-10-positive (IL-10^+^), anti-inflammatory, and immunosuppressive phenotype. Such macrophages suppress the effector function of CD8-positive (CD8^+^) cytotoxic T lymphocytes, whereas genetic restriction of GABA synthesis in the B-cell lineage enhances the antitumor response and control of tumor growth [131]. In parallel, tumor-derived GABA limits dendritic cell and T-cell infiltration, partly through the β-catenin-driven axis involving the chemokines C-C motif chemokine ligand 4 and 5 (CCL4/CCL5), creating a TME with a “non-T-cell-inflamed” phenotype [34].

Collectively, GABAergic signaling can generate calcium-restraining or calcium-promoting configurations, while its final phenotypic consequence may additionally be modified by downstream oncogenic and immune signaling [34,116,117,132]. A calcium-restraining configuration is expected when GABA_A_ hyperpolarizes the membrane under low intracellular Cl^−^ conditions and the relevant Ca^2+^-dependent programs remain sensitive to reduced membrane excitability [25,26,120,122]. Conversely, a calcium-promoting configuration can emerge when Cl^−^ accumulation renders GABA_A_ depolarizing and the target cell expresses Ca^2+^ channels capable of translating this depolarization into increased Ca^2+^ influx [117,121]. GABA_B_-dependent activation of proliferative or immunosuppressive pathways through GSK-3β/β-catenin represents a distinct downstream rewiring mechanism and should not be interpreted as a simple reversal of its classical Ca^2+^-inhibitory action [34].

Consequently, GABA in the TME should be understood not as an intrinsically inhibitory or excitatory mediator, but as a context-dependent regulator of Ca^2+^ response topology. Its calcium-regulatory effect is shaped by ligand exposure, receptor composition, chloride-transporter balance, calcium-channel repertoire, and the cellular compartment receiving the input, whereas downstream oncogenic and immune pathways remain additional determinants of phenotype. This provides the basis for treating GABAergic modulation as an integral component of neurochemical Ca^2+^ regulation rather than as an isolated inhibitory pathway.

### 2.4. The α2δ-1 Subunit as a Regulator of Ca^2+^ Response Amplitude in the Tumor Microenvironment

The α2δ-1 subunit of voltage-gated calcium channels regulates the availability and functional capacity of Cav1.x (L-type) and Cav2.x (particularly Cav2.1 and Cav2.2) Ca^2+^ channels, directly influencing the intensity of calcium ion influx into the cell [27,133,134]. Under physiological conditions, α2δ-1 functions as an auxiliary protein that enhances channel trafficking to the cell membrane, increases membrane expression and functional availability, and potentiates the coupling between Ca^2+^ influx and effector processes such as neurotransmitter release [52,53,133]. Consequently, α2δ-1 regulates Ca^2+^ response gain: it does not initiate the signal, but sets its amplitude, duration, and capacity to activate Ca^2+^-dependent pathways [27,133]. In this setting, α2δ-1 is not an independent regulatory input, but a threshold-setting parameter that determines whether α7-driven or depolarizing GABA_A_-driven Ca^2+^ signals become sufficient to activate proliferative, migratory, and repair programs.

In cancer, this function undergoes reprogramming and expansion. Overexpression of α2δ-1 leads to increased recruitment of functional Ca^2+^ channels to the cell membrane, enhanced Ca^2+^ influx, and stabilization of pro-proliferative activity [28,29,30]. This has been particularly well documented in NSCLC, where the α2δ-1-high cell subpopulation exhibits increased Ca^2+^ current through L-type and N-type channels, enhanced MAPK/ERK pathway activity, and cancer stem cell (CSC) features, including self-renewal capacity and sphere-forming ability [29,30]. Importantly, cells with high α2δ-1 expression also demonstrate increased efficiency of DNA damage repair following irradiation. This includes more rapid resolution of phosphorylated histone H2AX (γH2AX) foci and enhanced activation of ataxia-telangiectasia mutated (ATM) kinase, indicating that elevated Ca^2+^ influx directly supports mechanisms of radioresistance [29]. Analogous observations have been made in hepatocellular carcinoma (HCC), where α2δ-1 was identified as a marker of tumor-initiating cells; its expression correlates with enhanced Ca^2+^ influx, ERK1/2 activation, and increased tumorigenicity [28]. Individual clinicopathological studies indicate that α2δ-1 overexpression is a recurring phenomenon across multiple solid tumors—including non-small cell lung cancer, hepatocellular carcinoma, breast cancer, and ovarian cancer—and is consistently associated with poorer prognosis, an aggressive phenotype, and treatment resistance, while its level remains strongly dependent on tissue context [28,29,30,135,136]. Transcriptomic and immunological data from breast cancer further suggest that *CACNA2D1* expression may contribute to shaping the TME by modulating interactions with the immune system, including immune-checkpoint expression and the composition of cellular infiltrates, although the mechanisms underlying these associations require further validation [135].

α2δ-1 is therefore not merely a marker: it can determine whether a neurochemical stimulus remains subthreshold or becomes a biologically effective Ca^2+^ response [28,30]. High α2δ-1 expression increases the capacity to generate strong, sustained Ca^2+^ signals in response to stimuli that would remain weak in low-α2δ-1 cells. It can amplify both α7-driven Ca^2+^ entry and depolarizing GABA_A_ effects in cells with a reversed chloride gradient. Its effect is gain-setting rather than direction-setting.

Pharmacological modulation of α2δ-1 adds a translational dimension. In the nervous system, α2δ-1 ligands, including pregabalin and gabapentin, do not directly reduce either α2δ-1 protein synthesis or its total membrane abundance. They act through competitive antagonism at the L-amino acid binding site of α2δ-1, blocking its interaction with synaptic adaptor proteins, including thrombospondins and components of the active zone complex, thereby impairing the selective trafficking of α2δ-1 to active presynaptic zones. This reduces the density of functional Ca^2+^ channels in the presynaptic plane. The pool of channels within non-synaptic and somatic membranes may remain partially preserved [52,53,134,137]. This has direct implications for oncology: pregabalin would be expected to act primarily on α2δ-1-dependent trafficking in tumor-innervating synaptic terminals, rather than on the total tumor-cell channel pool, thereby altering local neurotransmitter availability through reduced presynaptic release.

This creates an asymmetry between the mechanistic target and the available pharmacological probe [30,53]. The relevant gain function resides in the tumor-cell-autonomous α2δ-1 pool, whereas gabapentinoids predominantly affect trafficking and synaptogenic α2δ-1 at innervating presynaptic terminals [30,53,137]. Pregabalin is therefore an indirect and partial probe of cell-autonomous gain: any oncological effect would combine altered tumor-cell channel availability with reduced presynaptic neurotransmitter release, and these components need not act in the same direction [30,53]. The clean test of the cell-autonomous gain is consequently genetic—*CACNA2D1* knockdown or overexpression in tumor cells, rather than pharmacological, and gabapentinoid sensitivity should be read as a composite output, not as a direct measure of tumor-cell α2δ-1 function [29,30,53]. Despite potential significance, these interactions remain hypothetical, as systematic in vivo studies of chronic α2δ-1 modulation in tumor progression are lacking [30,138]. α2δ-1 represents both a biologically justified therapeutic target in oncology and a stratification parameter for studies evaluating neurochemical interventions within the TME [28,29,30].

### 2.5. Hydrodynamic Constraints on Neurochemical Ca^2+^ Regulation

The tumor microenvironment is not a static biochemical space in which ligands diffuse freely toward receptors [36,37]. Its hydromechanical properties are expected to shape neurotransmitter concentrations within the interstitial space and thereby condition local Ca^2+^ dynamics [36,37,40]. Its physical architecture is therefore an active regulatory parameter, not merely a background feature [36,37].

The mechanistic basis of this phenomenon is the pathological remodeling of fluid transport within the TME, a consequence of abnormal angiogenesis [139]. Accordingly, solid tumors exhibit chaotic, permeable vessels lacking proper pericyte control, resulting in massive extravasation of fluid from the vascular bed into the interstitial space [139]. Simultaneously, the intratumoral lymphatic network is functionally impaired or entirely absent, preventing drainage of excess fluid [36]. The net effect is elevated interstitial fluid pressure (IFP), which in solid tumors greatly exceeds physiological values and strongly reduces local gradients driving convection, effectively equalizing interstitial and vascular pressure [36,38]. Consequently, diffusion becomes the dominant, yet spatially restricted, mechanism of molecular transport, and substances secreted locally by TME cells accumulate in the immediate vicinity of their source, forming locally sustained concentration gradients incapable of persisting in tissues with normal perfusion and drainage [37,140].

For neurochemical Ca^2+^ signaling, this mechanism is critical [19,36]. ACh and GABA, however, follow different clearance kinetics, which the spatial argument must account for [75,141]. GABA is removed chiefly by cellular uptake rather than by rapid extracellular degradation, so reduced convection directly prolongs its local persistence [140,141]. Acetylcholine is additionally subject to fast enzymatic hydrolysis by acetylcholinesterase (AChE) and butyrylcholinesterase (BuChE); the lifetime of a local ACh gradient therefore depends not on convection alone, but on local cholinesterase activity [76,136]. In niches with diffusion-limited clearance and reduced AChE/BuChE activity, ACh may reach higher and more persistent local concentrations capable of enhancing α7 engagement, whereas preserved cholinesterase activity would constrain this effect [142,143,144]. Local cholinesterase status therefore becomes a testable determinant of where α7-driven Ca^2+^ signaling can dominate [142]. GABA synthesized by B cells and macrophages within the TME does not disperse uniformly but may form local zones of elevated concentration within microsegments characterized by particularly impaired circulation [37,45,131,145]. Neither neurotransmitter therefore acts within the TME as a homogeneous, diffusively mixed signal. Instead, they are expected to generate spatial concentration gradients in which individual tumor cells encounter distinct ligand conditions depending on their distance from the source, the local density of the extracellular matrix (ECM), and the degree of drainage impairment [38,39,146].

Cumulative activation of nAChRs and GABAergic receptors depends on local ligand concentration and exposure duration, which together determine Ca^2+^ influx amplitude and the probability of secondary activation of intracellular stores [144,147]. Based on these transport constraints, cells located near local ACh sources in regions of impaired flow would be expected to experience higher and more prolonged ACh exposure, potentially altering α7 activation-desensitization dynamics [40,46]. Cells located in regions of more effective clearance may instead experience lower or shorter-lasting exposure, potentially generating a distinct calcium response [46,144]. Thus, within a single tumor, microregions exposed to distinct α7 activation-desensitization regimes may coexist with regions in which α4β2-linked or GABAergic modulation remains functionally prominent. This heterogeneity may arise from interstitial transport properties rather than from differences in receptor expression or α2δ-1 levels alone [37,46]. Consequently, the physical environment acts as an additional dimension of biological stratification, superimposed upon the molecular profile of individual cells. Heterogeneity of Ca^2+^ responses within a single tumor is therefore partially unavoidable: it arises not only from molecular instability of the cells, but from the physics of the interstitial space, which cannot be eliminated through manipulation of the expression of any single receptor [37,46,145].

This perspective has a direct methodological consequence: results obtained in vitro, where ligand kinetics are determined solely by medium concentration and free diffusion, are unlikely to accurately reflect the conditions occurring in vivo, even with identical receptor and channel expression profiles [143,145]. The interstitial environment is therefore not merely contextual; it is a spatial determinant of neurochemical Ca^2+^ regulation.

The mechanistic observations reviewed above originate from heterogeneous experimental systems and differ substantially in the degree to which they directly connect neurotransmitter signaling to calcium regulation in cancer. To distinguish established experimental findings from their subsequent integration into the proposed framework, Table 1 summarizes the principal published studies, their experimental context, measured calcium-relevant outputs, and the extent to which each observation supports, qualifies, or challenges the integrated interpretation developed below.

## 3. Integrated Model

CAGE integrates the preceding mechanisms into a state-based model of neurochemical Ca^2+^ regulation in the TME. It assigns four roles: α7 nAChR as a multimodal pro-calcium input, α4β2 nAChR as a putative cholinergic-GABAergic coupling node, GABA_A_/GABA_B_ receptors as direction-setting modulators, and α2δ-1 as the gain regulator [30,72,116]. Ca^2+^ is treated here as the downstream integrator through which receptor functional state, response gain, and spatial exposure are translated into distinct calcium-signaling states that can influence proliferation, migration, angiogenesis, or immunomodulation. The cholinergic module defines the initial input: α7 provides a direct Ca^2+^-permeable component, whereas α4β2 may couple cholinergic stimulation to GABA-dependent modulation. Their relative functional contribution is exposure-dependent, adding a temporal dimension to the receptor-balance component of the CAGE state. The GABAergic module then shapes calcium-signal direction: GABA_A_ depends on chloride-gradient polarity, whereas the classical GABA_B_ component restricts Cav2.x-dependent Ca^2+^ entry; GABA_B_-associated AKT-GSK-3β-β-catenin signaling is treated separately as a downstream rewiring mechanism rather than as a calcium-direction determinant [34,120,130]. The third layer, α2δ-1, sets response gain: it does not determine signal direction, but controls whether α7- or depolarizing GABA_A_-driven Ca^2+^ elevations remain subthreshold or become strong enough to activate proliferative, reparative, and radioresistance programs [29,30]. Finally, the spatial layer adds a fourth constraint: impaired drainage and diffusion-dominated transport are expected to generate spatially heterogeneous and locally sustained ACh and GABA exposure across tumor space [46,143].

Consequently, cells located in different TME microsegments encounter distinct ligand histories, potentially generating spatial heterogeneity of Ca^2+^ responses despite similar receptor-expression profiles. Taken together, the published observations summarized above suggest that these variables may combine into recurrent functional configurations. For the purpose of generating testable predictions, two principal calcium-regulatory states and one downstream rewiring condition are distinguished here.

1. A pro-excitatory state: α7-associated pro-calcium signaling, high α2δ-1 gain, and permissive or depolarizing chloride handling favor amplified Ca^2+^ responses.
2. A buffered state: preserved GABAergic restraint and hyperpolarizing chloride handling favor reduced or transient Ca^2+^ responses.
3. A rewired configuration represents a boundary condition rather than a third calcium-polarity state. It arises when downstream oncogenic or immune pathways modify the biological consequence expected from proximal neurochemical Ca^2+^ regulation. GABA_B_-associated PI3K/AKT-GSK-3β-β-catenin signaling provides one experimentally supported example of such rewiring [34].

**Figure 2:**
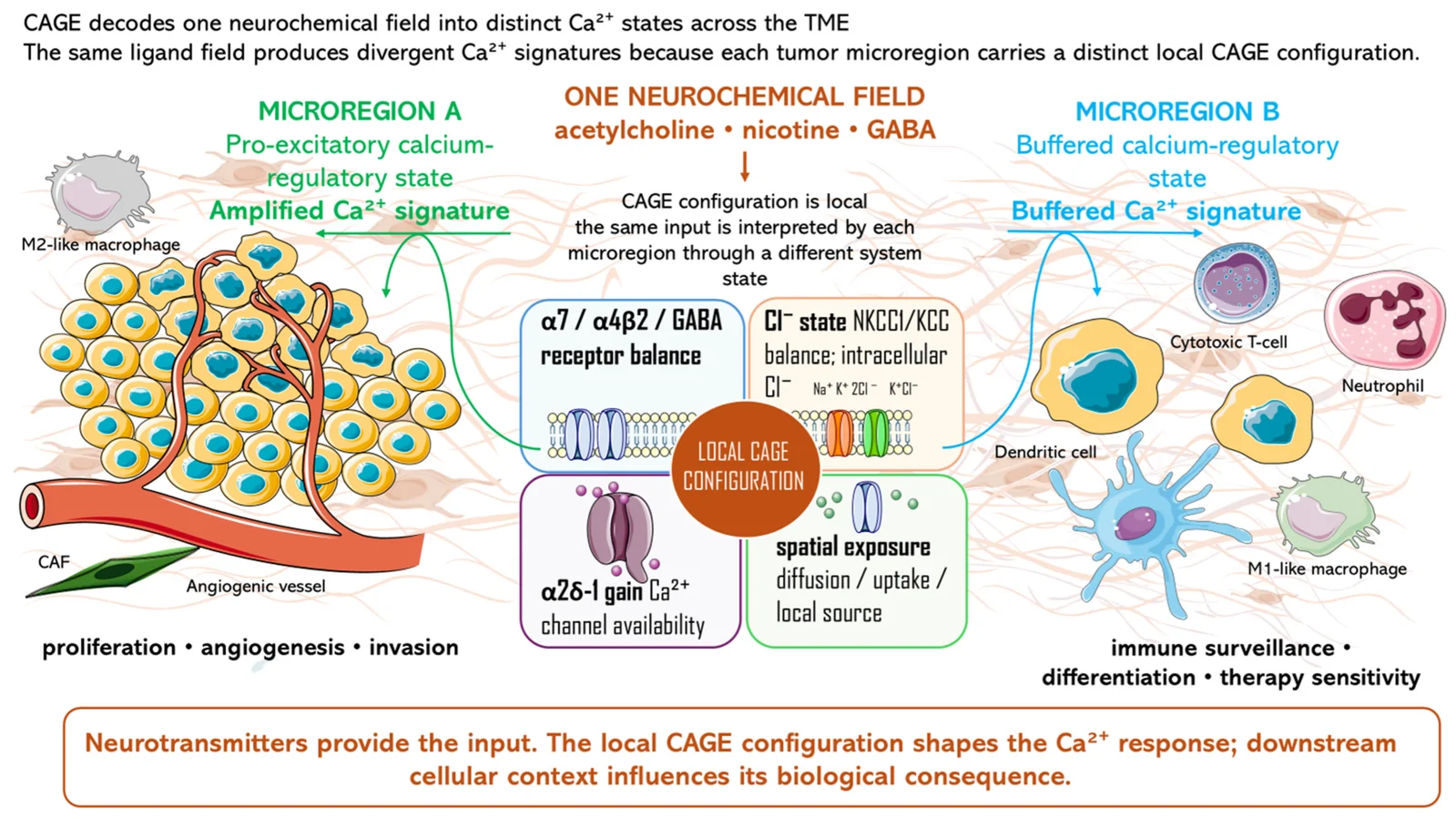
**Local decoding of a shared neurochemical field into divergent Ca^2+^ states across the TME.** A single neurochemical field composed of acetylcholine, nicotine-related cholinergic stimulation, and GABAergic signaling may give rise to distinct Ca^2+^ signatures in different tumor microregions. This divergence reflects local differences in receptor balance, chloride-gradient state, α2δ-1-dependent calcium gain, and spatial neurotransmitter exposure. In one tumor microregion, these variables may favor an amplified Ca^2+^ state associated with proliferation, angiogenesis, and invasion. In another tumor microregion, the same ligand field may instead be decoded into a buffered Ca^2+^ state associated with immune surveillance, differentiation, and therapy sensitivity. Thus, neurotransmitters provide the input, whereas the local configuration of the calcium-regulatory axis shapes the resulting Ca^2+^ signature; downstream cellular state further influences its biological consequence. Graphical elements from Servier Medical Art.

The calcium-regulatory states are not tumor subclasses, but local and potentially reversible configurations that may coexist within a single tumor and shift as receptor functional state, chloride handling, α2δ-1 gain, or ligand exposure changes locally. Downstream rewiring may coexist with either calcium-regulatory state and modify interpretation of the resulting biological phenotype rather than defining Ca^2+^ polarity itself. Whereas Table 1 summarizes the published evidence underlying individual mechanistic components, Table 2 presents their integrative organization as an operational matrix distinguishing the two calcium-regulatory states from downstream rewiring and linking these configurations to their experimental readouts. Their potential coexistence across spatially distinct tumor microregions is illustrated in Figure 2.

Integration of these elements shifts the unit of analysis from individual neurotransmitters to local calcium-axis profiles defined by receptor composition and functional state, chloride-gradient status, α2δ-1 gain, and spatial ligand exposure. These profiles provide a basis for functional tumor stratification across molecular and microregional parameters, rather than receptor expression alone. Therapeutically, this implies that interventions targeting α7, GABA_A_/GABA_B_ receptors, or α2δ-1 are unlikely to be interpretable without prior stratification of the local calcium-axis profile. The proposed model not only integrates observations that have thus far been described separately but also generates a set of falsifiable predictions, described in detail in the following section, that define specific directions for experimental validation of this concept.

## 4. Falsifiable Predictions

The integrated architecture generates predictions that are not reducible to isolated receptor effects and are amenable to experimental falsification. They link component-level perturbations to measurable Ca^2+^ outputs and biological consequences. Each prediction tests a defined mechanistic assumption and can therefore support, refine, or falsify the proposed architecture.

First, the relationship between cholinergic stimulation and Ca^2+^ output is predicted to depend jointly on agonist concentration and exposure duration. α7 and α4β2 nAChRs differ in both agonist sensitivity and desensitization behavior: homomeric α7 receptors exhibit exceptionally rapid desensitization kinetics, whereas α4β2 receptors are markedly sensitive to low nicotine concentrations and may enter long-lasting desensitized states during sustained exposure [113,114]. Consequently, identical extracellular concentrations of ACh or nicotine may produce different functional α7/α4β2 balances following acute versus prolonged stimulation, resulting in different Ca^2+^ and GABAergic outputs. The relationship between cholinergic input and calcium response is therefore expected to be time-dependent and potentially non-monotonic rather than governed by a fixed concentration threshold separating α7- and α4β2-dominated signaling. This prediction can be tested by systematically varying agonist concentration and exposure duration while pharmacologically resolving the α7- and α4β2-dependent components. PNU-282987 can be used for selective α7 activation [149], whereas α4β2 signaling can be interrogated using sazetidine-A, which strongly modulates α4β2 receptor function [147], together with the α4β2-preferring antagonist dihydro-β-erythroidine (DHβE) [73]. Simultaneous measurements of intracellular Ca^2+^ and extracellular GABA would determine whether the relative contribution of the two receptor-dependent components changes with exposure history [108,109]. Genetically encoded GABA and ACh sensors, combined with Ca^2+^ imaging, provide a direct approach for resolving these concentration- and time-dependent responses in real time [150,151].

Second, changes in cholinergic activity within the TME should be associated with changes in local GABAergic tone in a manner dependent on the dominant nAChR subtype, rather than on general cholinergic activity per se [73,109]. Selective α4β2 blockade should reduce the α4β2-dependent component of cholinergic-GABAergic coupling and alter its relationship with Ca^2+^ dynamics, whereas α7 blockade should preferentially suppress the direct pro-calcium component while allowing the residual α4β2-dependent contribution to GABAergic modulation to be assessed [108,147]. This can be tested in co-culture models containing GABA-producing immune cells, with real-time spatial imaging of neurotransmitter concentrations [131,150].

Third, the effect of GABA on Ca^2+^ should reverse sign depending on the status of chloride transporters in the target cell: in cells with high NKCC1 activity and relative functional deficiency of KCC1/KCC3, GABA_A_ activation should lead to a paradoxical increase in Ca^2+^ influx, whereas pharmacological inhibition of NKCC1 with bumetanide should restore the hyperpolarizing and thereby antiproliferative effect of GABA_A_ [25,26,117,121,152]. This assumption is experimentally accessible in cell lines with differential NKCC1 expression, for example, by comparing TNBC cells with high NKCC1 expression to lines with low expression, with membrane potential measurements using patch-clamp methodology and parallel Ca^2+^ imaging [25,117].

Fourth, α2δ-1 modulation should scale the Ca^2+^ response to both pro-calcium stimuli (α7 activation) and depolarizing cues (GABA_A_ in cells with a reversed Cl^−^ gradient), without altering the direction of the effect [27,133]. This prediction can be tested in NSCLC cells with *CACNA2D1* knockdown or overexpression by measuring the Ca^2+^ response to selective activation of individual transduction components while maintaining a comparable genetic background [29,30].

Fifth, heterogeneity of the Ca^2+^ response within the tumor should be partially mappable to the spatial distribution of neurotransmitters, rather than exclusively to differences in gene expression [46,153]. Tumor cells with identical transcriptomic profiles but localized in different microenvironmental zones, with differing IFP and varying distances from immune cells producing ACh or GABA, should exhibit systematically distinct calcium responses [46,131,154]. This prediction is testable through the integration of spatial transcriptomics (Visium, multiplexed error-robust fluorescence in situ hybridization [MERFISH]) with single-cell in situ Ca^2+^ imaging and mapping of TME microsegments using mass spectrometry imaging methods [155,156,157]. Testing these predictions requires tools that combine receptor profiling, ligand mapping, and Ca^2+^ readouts at the level of individual TME microsegments, a level of spatial resolution that standard in vitro models cannot provide [37,156].

## 5. Conceptual Implications for Tumor Biology and Cancer Neuroscience

Three conceptual implications follow from this proposed organization of neurochemical Ca^2+^ regulation in the TME. They change how research questions and experimental outcomes should be interpreted in cancer neuroscience. The first implication is a change in the unit of analysis. The prevailing approach asks: “does a given neurotransmitter inhibit or stimulate proliferation?” This question produces inconsistent answers because it treats ligand identity as the primary explanatory variable. The relevant question becomes: What is the calcium-axis profile of a given TME? Accordingly, divergent effects of ACh, nicotine, and GABA should be tested against explicitly measured differences in calcium-axis configuration rather than attributed to ligand identity alone [9,34]. This shift is not semantic: it converts apparent contradictions into testable comparisons. If divergent outcomes persist despite matched calcium-axis configurations, they would indicate either downstream biological determinants outside the present framework or a failure of one of its mechanistic assumptions.

The second implication is that the physical TME is a biological variable, not merely a context. The spatial distribution of ligands, shaped by elevated IFP, leaky vasculature, and impaired lymphatic drainage, is expected to influence the duration of cellular exposure to ACh and GABA, and thereby the amplitude and persistence of the Ca^2+^ response [143,145]. Thus, in vitro results, where ligand kinetics are determined by medium concentration and free diffusion, may systematically fail to reflect in vivo conditions even when receptor and channel expression profiles are matched [38,144]. Pharmacological studies that ignore ligand distribution and transport variables in the TME may therefore misestimate both response direction and effective concentration ranges [39]. Consequently, half-maximal inhibitory concentration (IC_50_) and half-maximal effective concentration (EC_50_) values determined in vitro for nAChR and GABA receptor ligands may differ systematically from the effective in vivo concentrations. This has direct implications for dose selection in preclinical and clinical studies [145,158].

The third implication concerns therapeutic stratification. Interventions targeting α7 nAChR, GABA_A_/GABA_B_ receptors, or α2δ-1 will be interpretable only against the functional calcium-axis profile of the target tumor, including NKCC1/KCC1/KCC3 balance, Cl^−^ gradient status, α2δ-1 level, and dominant neurotransmitter source within the TME [25,26,30,122]. The same intervention may produce divergent, even opposite, effects across cancer types, and calcium-axis profiling can test whether differences in neurochemical Ca^2+^ regulation contribute to this variability [9]. At minimum, stratification should include α7 receptor composition, including *CHRNA7/CHRFAM7A* status, α4β2, GABA_A_/GABA_B_, α2δ-1, NKCC1, and Cl^−^ gradient status in trials evaluating neurochemical interventions in oncology. The absence of such stratification may have contributed to variability in previous studies involving nAChR ligands, although this possibility requires prospective validation [9].

## 6. Limitations and Key Areas for Validation

Several limitations define the key areas in which the present framework requires experimental validation.

The most significant limitation is the lack of direct functional data confirming α4β2 and GABA coupling in cancer cells or TME cells other than neurons. All currently available data describing this mechanism derive from neuronal models, specifically hippocampal and cortical interneuron preparations, whose presynaptic architecture and Cav2.x channel repertoire differ fundamentally from the conditions present in tumor cells lacking a classical neuronal presynaptic organization [9,73,109]. Extrapolation to the TME is biologically justified, yet it remains a hypothesis requiring verification in oncological in vitro and/or in vivo models. Experimental verification of this mechanism should therefore be considered a priority for future research, as without such validation, the α4β2 coupling node remains the least well-documented component of the model [9,73]. Failure to detect functional α4β2-dependent modulation of GABAergic output in appropriately selected oncological models would restrict or invalidate this coupling node as a TME component of the framework, even if the broader calcium-centered architecture remained applicable. A further limitation is that receptor-expression measurements alone cannot define the functional nAChR state. More broadly, validation should distinguish prediction of the calcium-regulatory response from prediction of the final biological phenotype, because downstream oncogenic and immune pathways may modify the consequence of any given Ca^2+^ state.

The second limitation arises from TME heterogeneity: both the expression of relevant components (α7, α4β2, GABA_A_, GABA_B_, α2δ-1, NKCC1, KCC1/KCC3) and the cellular composition determining local neurotransmitter sources differ among cancer types, disease stages, and individual tumors/tumor loci [9,29,30,122,135,136]. This complicates the direct translation of the model into clinical predictions without prior molecular stratification of the calcium axis profile of a given tumor [9]. A practical validation strategy is to integrate spatial transcriptomics with metabolomic analysis of tumor interstitial fluid (TIF), enabling simultaneous mapping of receptor expression and local ligand concentrations within the same TME microsegment [37,155,157].

The third limitation is the lack of methods for precise in vivo measurement of neurotransmitter concentrations in tumor interstitial space at the spatial resolution required to test ligand-gradient effects [37,156]. Genetically encoded neurotransmitter indicators (GENIs), including GACh sensors for ACh and iGABASnFR for GABA, represent one of the most promising tools for this purpose; however, their application to in vivo models of solid tumors remains technically limited and has not yet been widely adopted in cancer biology [150,151]. Adaptation of these biosensors to TME conditions, including addressing challenges related to vector delivery, tissue autofluorescence, and signal stability under hypoxic and acidic conditions, is a key methodological requirement for validating ligand-gradient effects [65,150,151].

The fourth limitation concerns selective pharmacological modulation of individual nAChR subtypes in vivo. This constitutes a prerequisite for testing the model’s predictions in complex systems. Existing selective ligands, including PNU-282987 for α7 and sazetidine-A for α4β2, exhibit sufficient selectivity for in vitro models, but their systemic application is limited by CNS receptor distribution and potential neurological effects [147,149,159]. For α7, an additional methodological challenge is that receptor expression and ion-channel activity alone may not fully capture signaling competence, because ionotropic conductance and metabotropic intracellular signaling can represent partially dissociable receptor outputs [89,90,94]. Experimental validation should therefore distinguish these signaling modes when assigning functional α7 states. Further application of these ligands in long-term in vivo cancer models requires pharmacokinetic optimization or the development of TME-targeted delivery strategies, for example, through conjugation with ligands specific to tumor cells.

Collectively, these limitations define four closely interconnected research priorities: (1) functional verification of α4β2 and GABA coupling in cellular and in vivo models of lung and pancreatic cancer; (2) characterization of calcium-axis molecular profiles in different cancer subtypes as a prerequisite for stratification; (3) application of modern techniques for imaging neurotransmitter gradients in the TME in vivo; (4) development of strategies for selective modulation of nAChR subtypes with limited systemic effects. Progress in these areas will either validate or falsify the framework while generating tools and data of broader relevance to TME biology and cancer neuroscience.

## 7. Summary of the Model

Despite these limitations, the value of the model lies in making its assumptions experimentally testable rather than leaving them implicit. Organizing the accumulating neurochemical evidence around a single Ca^2+^ effector offers a route from fragmented correlations toward testable, predictive cancer neuroscience. Understanding the neurochemical configuration of the calcium axis may provide a basis for molecular stratification and for therapeutic strategies that consider Ca^2+^ as an integrated effector rather than any single receptor in isolation. This shift proposes the calcium-regulatory state of the tumor microenvironment as a more informative unit of analysis than receptor or ligand identity alone. Such a state is expected to constrain neurochemically driven Ca^2+^ responses, while downstream oncogenic and immune programs remain additional determinants of the final biological phenotype.

## 8. Methodology

Relevant literature was identified through searches of PubMed, Scopus, and Web of Science, without restrictions on publication date. Only openly accessible sources were consulted; no subscription-based or institutionally licensed content was included.

Search terms included tumor microenvironment, calcium signaling, nicotinic acetylcholine receptors, GABAergic signaling, α2δ-1, CACNA2D1, and cancer neuroscience, applied individually and in combination to capture literature on cholinergic and GABAergic receptor signaling, α2δ-1-dependent channel regulation, and their convergence on calcium dynamics within the tumor microenvironment.

Approximately 350 articles were read in full. Of these, 159 were retained in the final reference list, selected on the basis of mechanistic specificity and direct relevance to the conceptual model presented in this review. Primary research articles were favored over reviews where available. Studies limited to general nAChR or GABA receptor pharmacology without a calcium-signaling component, or to α2δ-1/voltage-gated calcium channel biology outside oncological contexts, were not prioritized for inclusion.

Source selection and evaluation were performed manually by the authors, without a formal registered protocol or automated screening tool, based on scientific reliability, mechanistic clarity, and relevance to the proposed framework.

Generative artificial intelligence (GenAI) was not used in literature screening, source selection, writing, or figure generation.

## Figures and Tables

**Table 1:** Published experimental evidence relevant to the mechanistic components integrated in the proposed framework.

Experimental Model/Published Study	Perturbation/Exposure	Transmitter/Cholinergic Change	GABAergic/Ionic Change	CaV/Ca^2+^ Readout	Downstream Signaling/Biological Output	Evidence Relative to the Proposed Framework	Refs.
NSCLC cells (A549, H1299) and tumor models	Nicotine/NNK; α7 blockade or CHRNA7 knockdown	α7-dependent nicotinic signaling ↑; effects reduced by α-bungarotoxin or CHRNA7 knockdown	─	Intracellular Ca^2+^ ↑ following α7 stimulation	PI3K/AKT and MAPK/ERK activation; cyclin D1 ↑; proliferation/tumor growth ↑	Supports: direct oncological evidence linking α7 activation to increased Ca^2+^ signaling and protumor output	[20,33,99]
Breast cancer cells (MCF-7)	Choline/nicotine; pharmacological Gα_i/o_ or Gα_q_ interference; disruption of α7 G-protein-binding region	α7 activation and α7–heterotrimeric G-protein coupling	─	Intracellular Ca^2+^ signaling ↑; G-protein interference attenuates the response	Proliferation and motility ↑	Supports and extends: demonstrates a metabotropic, calcium-relevant α7 output in cancer cells in addition to ionotropic conductance	[102]
Tumor-associated macrophages in CRC models	α7 knockdown/pharmacological α7 inhibition in macrophages	α7 signaling in TAMs ↓	─	Not directly measured	JAK2/STAT3 signaling altered; loss of macrophage α7 increases CRC-cell migration/invasion and is associated with metastatic behavior	Qualifies: α7 is not uniformly protumor across TME compartments; receptor-associated phenotype depends on the responding cell type	[104]
Metastatic melanoma; α7 versus α7/dupα7 receptors	Nicotine; recombinant SLURP-1; Oncotag	α7/dupα7 hybrid channels remain functional; nicotine potency not significantly altered by dupα7 incorporation	─	Functional receptor currents retained; tumor-cell Ca^2+^ was not the principal endpoint	Hybrid receptors show reduced sensitivity to recombinant SLURP-1, whereas Oncotag retains similar inhibitory efficacy; high CHRFAM7A/dupα7 is associated with recombinant SLURP-1 resistance	Qualifies: receptor abundance alone does not define α7 functional state; subunit composition changes ligand-specific responses without uniformly abolishing α7 function	[87]
A431 epidermoid carcinoma cells and xenografts	Recombinant SLURP-1/Oncotag	Negative allosteric modulation of α7; full-length SLURP-1 can additionally interact with EGFR	─	Direct Ca^2+^ flux was not the principal endpoint	Multiple mitogenic pathways inhibited; proliferation, xenograft growth and metastatic progression reduced	Qualifies: endogenous-like extracellular regulation modifies α7-associated oncological output independently of receptor abundance	[86,88]
Rat hippocampal/neuronal GABAergic preparations	Acute ACh/choline/nicotinic stimulation; DHβE blockade	Presynaptic α4β2 activation ↑	GABA release ↑; effect sensitive to α4β2-preferring antagonism	Presynaptic Cav2.1/Cav2.2-dependent Ca^2+^ entry contributes to GABA exocytosis	Increased inhibitory GABAergic transmission	Supports mechanistic node but limits generalization: provides direct evidence for α4β2-GABA coupling in neuronal preparations, but the mechanism has not yet been demonstrated in cancer/TME cells	[22,73,108,109,110]
NNK-induced pulmonary and pancreatic adenocarcinoma/NCI-H322-related models	Chronic NNK/nicotine exposure	α7 and α4-containing nAChR protein expression ↑; stimulatory nicotinic signaling ↑	GAD65 ↓; GABA ↓	Not directly measured	cAMP-associated stimulatory signaling ↑; inhibitory GABAergic tone ↓	Supports and qualifies: demonstrates oncological cholinergic-GABAergic interaction, but receptor expression cannot be equated with functional receptor activity or desensitization state	[111,112,115]
TNBC cells (HCC1806, BT-549)	GABA_A_ receptor blockade/β3-subunit knockdown	─	Functional β3-containing GABA_A_ receptors and Cl^−^ conductance demonstrated; blockade disrupts this axis	Ca^2+^ was not directly established as the causal intermediate	Cyclin D1 ↓, p21 ↑, G_0_/G_1_ arrest; proliferation and migration ↓	Supports with a mechanistic gap: shows cancer-cell dependence on GABA_A_/Cl^−^ signaling, but the predicted Cl^−^→membrane potential→Ca^2+^ sequence still requires direct Ca^2+^ measurement	[117]
BRAF^V600E^ melanoma-initiation model	Disruption of endogenous GABAergic melanoma–keratinocyte communication	─	GABA-dependent bioelectrical communication requires chloride conductance	Direct Ca^2+^ coupling was not resolved	Disruption of the GABAergic circuit weakens melanoma initiation	Supports with a mechanistic gap: provides in vivo-relevant evidence for chloride-dependent GABAergic tumor regulation, without directly establishing the downstream Ca^2+^ step	[125,126]
Lung/colorectal cancer models with tumor-derived GABA	Increased GAD1-dependent GABA production/GABA_B_ signaling	─	Tumor-derived GABA ↑; autocrine GABA_B_ signaling engaged	Classical Cav2.x inhibition was not the measured determinant of the phenotype	GSK-3β inhibitory phosphorylation ↑; β-catenin stabilization ↑; cyclin D1 ↑; proliferation ↑; reduced immune-cell infiltration/immunosuppression	Supports downstream rewiring: demonstrates that GABA_B_-associated oncogenic signaling can modify phenotype independently of a simple “GABA_B_ = Ca^2+^ brake” interpretation	[34]
CRC cells (HT29; also HCT116)	GABA_B_ receptor activation with baclofen	─	GABA_B_ signaling ↑	Ca^2+^ not directly measured	GSK-3β activity ↓ and NF-κB signaling ↓; G_1_ arrest; proliferation ↓	Challenges a uniform phenotypic interpretation: similar proximal GABA_B_/GSK-3β regulation is associated with growth inhibition rather than the protumor output reported in other CRC models; the determinant of this divergence remains unresolved	[132]
CRC cells (RKO, LoVo), patient tissue and xenografts	GABBR1 knockdown	─	GABA_B_ R1 signaling ↓	Ca^2+^ not directly measured	Hippo/YAP1 activation ↑; proliferation, migration, invasion and EMT ↑; tumor growth ↑; higher GABA_B_ R1 expression associated with better outcome	Challenges/defines a boundary: supports a tumor-restraining role of GABA_B_ R1 in CRC and shows that downstream phenotype cannot be predicted from receptor identity alone	[148]
NSCLC α2δ-1-high tumor-initiating/stem-like cells	High α2δ-1 expression; CACNA2D1 manipulation	─	─	L- and N-type Ca^2+^ current ↑; functional Ca^2+^-channel availability ↑	MAPK/ERK activity ↑; self-renewal/sphere formation ↑; ATM-associated DNA-damage repair ↑; radioresistance ↑	Supports: direct oncological evidence that α2δ-1 acts as a calcium-response gain regulator associated with aggressive tumor-cell states	[29,30]
Hepatocellular carcinoma tumor-initiating cells	High α2δ-1 expression/α2δ-1 targeting	─	─	Ca^2+^ influx ↑	ERK1/2 activation ↑; tumor-initiating capacity and tumorigenicity ↑	Supports: independent cancer model linking α2δ-1-dependent calcium competence to tumorigenic output	[28]
B-cell/plasma-cell GABA in tumor models	Genetic restriction or preservation of B-cell-lineage GABA synthesis	─	B-cell-derived GABA promotes IL-10^+^ macrophage differentiation	Cancer-cell Ca^2+^ not directly measured	Immunosuppressive macrophage program ↑; CD8^+^ T-cell activity ↓; restriction of GABA synthesis improves antitumor control	Qualifies/defines downstream boundary: demonstrates a major GABA-dependent TME phenotype that does not require direct regulation of cancer-cell Ca^2+^	[131]
Tumor interstitial transport/fluid-distribution models	Elevated IFP, impaired lymphatic drainage, diffusion-dominated transport	Local ligand production retained; direct intratumoral ACh gradients not measured	Direct intratumoral GABA gradients not measured	Spatial Ca^2+^ consequences not directly measured together with neurotransmitter gradients	Restricted convection and local accumulation of soluble molecules demonstrated in tumors	Indirect support/unresolved prediction: establishes the physical basis for spatially heterogeneous ligand exposure, but direct spatial coupling of ACh/GABA gradients to Ca^2+^ states in tumors remains to be demonstrated	[37,38,39,40,140]

Note. “Supports” denotes evidence directly consistent with a mechanistic component of the framework; “qualifies” denotes evidence that supports the component while limiting its generalization; “challenges” denotes published findings that cannot be explained by receptor identity or proximal signaling alone and therefore define a current boundary of prediction. “Not directly measured” is stated explicitly where the cited study did not simultaneously quantify the indicated calcium-relevant variable. Downstream biological phenotypes are not used to define calcium-regulatory states. Published GABA_B_ receptor studies in colorectal cancer (CRC) provide a clear example of evidence that is not fully resolved by the present framework. While tumor-derived GABA has been linked to GABA_B_-dependent protumor signaling, independent studies report growth-restraining GABA_B_ effects in colorectal cancer [34,132,148]. The determinants of this divergence remain unresolved, and these findings therefore define a current predictive limitation rather than a context that can be assigned retrospectively to a predefined calcium-regulatory state. ↑ indicates an increase/enhancement; ↓ indicates a decrease/reduction.

**Table 2:** Configuration matrix of the CAGE axis in the tumor microenvironment.

CAGE Dimension	Pro-Excitatory Calcium-Regulatory State	Buffered Calcium-Regulatory State	Rewired/Downstream-Modified Configuration	Priority Readouts/Experimental Access
System identity	α7-dominant, high-gain decoding of the shared ligand field; pro-calcium architecture	GABA-modulated, low-gain decoding of the shared ligand field; calcium-buffered architecture	Downstream oncogenic or immune rewiring of calcium-state interpretation	Define local state rather than receptor presence alone.
Receptor balance	α7-associated pro-Ca^2+^ signaling dominant; α4β2-GABA coupling weak or functionally uncoupled	α4β2/GABA coupling preserved; GABA_A_/GABA_B_ tone functionally active	Receptor balance does not define rewiring; downstream oncogenic or immune pathway engagement modifies the consequence of receptor signaling	*CHRNA7/CHRFAM7A*, *CHRNA4/CHRNB2* and GABA_A_/GABA_B_ receptor mapping; ligand-response profiling under defined exposure conditions.
Chloride-gradient state	NKCC1 predominance; GABA_A_ may become depolarizing and pro-Ca^2+^	Cl^−^ extrusion preserved through KCC1/KCC3/AE/NBC activity; GABA_A_ hyperpolarizes and restricts VGCC opening	Not state-defining; rewiring may occur under either hyperpolarizing or depolarizing chloride conditions	NKCC1/KCC1/KCC3/AE/NBC expression; intracellular Cl^−^ and ECl measurements.
α2δ-1 gain/calcium competence	High α2δ-1; receptor activation efficiently reaches Ca^2+^-dependent thresholds	Low or moderate α2δ-1; identical input remains subthreshold or transient	Not state-defining; downstream rewiring may coexist with low or high calcium-response gain	*CACNA2D1*/α2δ-1 protein level; VGCC surface availability; pregabalin/gabapentin sensitivity.
Spatial neurotransmitter exposure	Local ACh/nicotine-related stimulation sustained by diffusion-limited clearance	Shorter exposure, stronger uptake or immune-accessible drainage conditions	Not state-defining; spatial exposure may modify proximal Ca^2+^ signaling without determining downstream rewiring	ACh/GABA spatial maps; source-cell proximity; IFP, lymphatic drainage and ECM density.
Ca^2+^ signature	Sustained or oscillatory high-amplitude Ca^2+^ response	Transient, low-amplitude or buffered Ca^2+^ response	Not uniquely defined; may coexist with amplified or buffered Ca^2+^ signaling	Live-cell Ca^2+^ imaging; amplitude, duration, frequency and spatial propagation.
Expected/associated biological output	Proliferation, angiogenesis, invasion and survival	Immune surveillance, differentiation and therapy sensitivity	Stemness, immune exclusion, metastatic niche support and adaptive resistance	Spatial transcriptomics, IHC, EMT/stemness markers, immune infiltration and vascular density.
Therapeutic implication	Block of a single ligand/receptor may fail without calcium-axis stratification	Preserving or restoring buffering tone may favor treatment sensitivity	Calcium-axis stratification alone may be insufficient when downstream rewiring dominates phenotype	Combine ligand perturbation with CAGE-state stratification before efficacy testing.

Note. Pro-excitatory and buffered states describe local and potentially reversible calcium-regulatory configurations rather than tumor subclasses. The rewired configuration denotes downstream modification of the biological consequence of neurochemical Ca^2+^ regulation rather than a third Ca^2+^-polarity state. Expected biological outputs are not used to define the configurations themselves.

## Data Availability

No new data were created.

## Abbreviations

The following abbreviations are used in this manuscript:

AChE	acetylcholinesterase
AE	anion exchanger
AKT	protein kinase B
ATM	ataxia-telangiectasia mutated kinase
Bax	Bcl-2-associated X protein
Bcl-2	B-cell lymphoma 2
BuChE	butyrylcholinesterase
CAF	cancer-associated fibroblast
CAGE	Calcium-Gated Excitability
*CACNA2D1*	calcium voltage-gated channel auxiliary subunit alpha-2/delta-1 gene
Cav	voltage-gated calcium channel
CCL4	C–C motif chemokine ligand 4
CCL5	C–C motif chemokine ligand 5
CD8	cluster of differentiation 8
ChAT	choline acetyltransferase
*CHRFAM7A*	CHRNA7 family with sequence similarity 7A gene
*CHRNA4*	cholinergic receptor nicotinic alpha 4 subunit gene
*CHRNA7*	cholinergic receptor nicotinic alpha 7 subunit gene
*CHRNB2*	cholinergic receptor nicotinic beta 2 subunit gene
CICR	calcium-induced calcium release
CNS	central nervous system
CRAC	calcium release-activated calcium
CRC	colorectal cancer
CSC	cancer stem cell
cAMP	cyclic adenosine monophosphate
DHβE	dihydro-β-erythroidine
ECM	extracellular matrix
EC_50_	half-maximal effective concentration
EGFR	chloride equilibrium potential
ECl	epidermal growth factor receptor
EMT	epithelial–mesenchymal transition
ER	endoplasmic reticulum
ERK	extracellular signal-regulated kinase
GABA	γ-aminobutyric acid
GABA_A_	γ-aminobutyric acid type A receptor
GABA_B_	γ-aminobutyric acid type B receptor
*GABBR1*	gamma-aminobutyric acid type B receptor subunit 1 gene
*GAD1*	glutamate decarboxylase 1 gene
GAD65	65 kDa glutamate decarboxylase isoform
GENI	genetically encoded neurotransmitter indicator
G_i/o_	inhibitory G-protein family
Gα_i/o_	inhibitory G-protein alpha subunit
Gα_q_	G-protein alpha q subunit
Gβγ	G-protein beta-gamma subunit complex
GSK-3β	glycogen synthase kinase-3 beta
HCC	hepatocellular carcinoma
HIF-1α	hypoxia-inducible factor 1 alpha
HUVEC	human umbilical vein endothelial cell
IC_50_	half-maximal inhibitory concentration
IFP	interstitial fluid pressure
IHC	immunohistochemistry
IL-10	interleukin 10
IP_3_	inositol 1,4,5-trisphosphate
IP_3_R	inositol 1,4,5-trisphosphate receptor
JAK	Janus kinase
KCC1	K^+^–Cl^−^ cotransporter 1
KCC2	K^+^–Cl^−^ cotransporter 2
KCC3	K^+^–Cl^−^ cotransporter 3
Ly6/uPAR	lymphocyte antigen 6/urokinase-type plasminogen activator receptor
MAPK	mitogen-activated protein kinase
MERFISH	multiplexed error-robust fluorescence in situ hybridization
mTORC2	mechanistic target of rapamycin complex 2
nAChR	nicotinic acetylcholine receptor
NBC	sodium–bicarbonate cotransporter
NF-κB	nuclear factor kappa B
NKCC1	Na^+^–K^+^–2Cl^−^ cotransporter 1
NNK	4-(methylnitrosamino)-1-(3-pyridyl)-1-butanone
NSCLC	non-small cell lung cancer
PI3K	phosphoinositide 3-kinase
PLC	phospholipase C
PKA	protein kinase A
*SLC12A2*	solute carrier family 12 member 2 gene
*SLC12A5*	solute carrier family 12 member 5 gene
SLURP-1	secreted Ly6/uPAR-related protein 1
SOCE	store-operated calcium entry
STAT	signal transducer and activator of transcription
STIM	stromal interaction molecule
TAM	tumor-associated macrophage
TIF	tumor interstitial fluid
TME	tumor microenvironment
TNBC	triple-negative breast cancer
TRP	transient receptor potential
VAChT	vesicular acetylcholine transporter
VGCC	voltage-gated calcium channel
γH2AX	phosphorylated histone H2AX
